# Towards Personalized Radiotherapy in Pelvic Cancer: Patient-Related Risk Factors for Late Radiation Toxicity

**DOI:** 10.3390/curroncol32010047

**Published:** 2025-01-17

**Authors:** Anna C. Nuijens, Arlene L. Oei, Nicolaas A. P. Franken, Coen R. N. Rasch, Lukas J. A. Stalpers

**Affiliations:** 1 Department of Radiation Oncology, Amsterdam UMC Location University of Amsterdam, Meibergdreef, 1105 AZ Amsterdam, The Netherlandsl.stalpers@amsterdamumc.nl (L.J.A.S.); 2 Laboratory for Experimental Oncology and Radiobiology (LEXOR), Center for Experimental and Molecular Medicine (CEMM), Amsterdam UMC Location University of Amsterdam, Meibergdreef, 1105 AZ Amsterdam, The Netherlands; 3 Cancer Center Amsterdam, Imaging and Biomarkers, Meibergdreef, 1105 AZ Amsterdam, The Netherlands; 4 Department of Radiation Oncology, Leiden University Medical Center, Albinusdreef, 2333 ZA Leiden, The Netherlands

**Keywords:** external beam radiotherapy, late radiation toxicity, quality of life, genetic predisposition, biomarkers, predictive factors, personalized treatment, prostate cancer, cervical cancer

## Abstract

Normal tissue reactions vary significantly among patients receiving the same radiation treatment regimen, reflecting the multifactorial etiology of late radiation toxicity. Predicting late radiation toxicity is crucial, as it aids in the initial decision-making process regarding the treatment modalities. For patients undergoing radiotherapy, anticipating late toxicity allows for planning adjustments to optimize individualized care. Various dosimetric parameters have been shown to influence the incidence of late toxicity, and the literature available on this topic is extensive. This narrative review examines patient-related determinants of late toxicity following external beam radiotherapy for pelvic tumors, with a focus on prostate and cervical cancer patients. In Part I, we address various methods for quantifying radiation toxicity, providing context for interpreting toxicity data. Part II examines the current insights into the clinical risk factors for late toxicity. While certain factors—such as previous abdominal surgery, smoking behavior, and severe acute toxicity—have consistently been reported, most of the others show inconsistent associations. In Part III, we explore the influence of genetic factors and discuss promising predictive assays. Single-nucleotide polymorphisms (SNPs) likely elevate the risk in specific combinations. Advances in artificial intelligence now allow for the identification of SNP patterns from large datasets, supporting the development of polygenic risk scores. These innovations hold promise for improving personalized treatment strategies and reducing the burden of late toxicity in cancer survivors.

## 1. Introduction

Radiotherapy is a cornerstone treatment modality for various pelvic tumors, including non-metastatic prostate cancer and locoregionally advanced cervical cancer. Technological advances in radiotherapy have allowed for tumor dose escalation, leading to improved tumor control, while exposure to the surrounding healthy tissues remains limited [1,2]. Despite these advancements, radiotherapy inevitably hits normal tissues, precipitating potential adverse effects. In external beam radiotherapy (EBRT) for prostate and cervical cancer, the bowel and bladder belong to the main organs at risk since these are unavoidably included in the radiation field. Consequently, examples of adverse effects include diarrhea, urinary frequency, abdominal pain, urinary or rectal blood loss, and incontinence. These adverse effects are typically divided into acute and late radiation toxicity, with an arbitrary boundary of three months after the completion of treatment [3]. Toxicities can take up many years to develop, but for practical evaluation purposes, most clinical studies report the crude incidence and/or prevalence rates at 2 to 3 years following treatment [4,5,6,7]. Historically, late toxicity was considered to be irreversible and progressive [3,8,9,10,11]. In the past, toxicity reporting thus focused on the (first) occurrence of moderate and severe events [3,7]. It is becoming apparent that toxicity can display a reversible pattern, and therefore in more recent studies, the duration of symptoms has also been addressed [12,13,14,15,16,17]. Depending on the severity and duration of a given symptom, it can severely affect quality of life (QoL).

Prostate cancer is the most common cancer in Dutch males; in men worldwide, it is the second most frequently diagnosed cancer [18,19]. It is likely that the number of patients with prostate cancer will rise further due to aging of the population and due to its early detection using prostate-specific antigen (PSA) screening. Most patients with non-metastatic prostate cancer can be effectively treated using EBRT, resulting in favorable long-term overall survival [19,20,21]. The majority of these men will live more than 10 years. Walz et al. reported a median life expectancy of 13.8 years for patients with localized disease treated using either EBRT or radical prostatectomy [22]. Due to its high incidence and survival rates, a large group of irradiated patients will develop complications like rectal bleeding, diarrhea, and urinary frequency or urgency. In approximately 30% of patients, symptoms are classified as moderate or severe late radiation toxicity, though the toxicity rates vary widely [23,24,25,26,27,28].

Cervical cancer represents a major global health challenge, ranking as the second most common cancer and a leading cause of mortality among women in low-Human Development Index (HDI) countries [29]. In contrast, it is relatively rare in high-HDI countries. For example, the Netherlands reported an incidence of approximately 9 cases per 100,000 women in 2020 [29,30]. The primary cause of cervical cancer is persistent infection with high-risk types of human papillomavirus (HPV), a common family of viruses transmitted via sexual contact. Vaccines protect against these high-risk HPV types, and screening programs can detect early signs of the disease, making cervical cancer one of the most preventable and treatable cancers. However, around 90% of both cases and death occur in low- and middle-HDI countries, where access to prevention and treatment is more limited. In high-HDI countries, mortality rates have consistently declined in recent decades, largely due to effective early detection and improved treatments [31]. Although surgery is the cornerstone of the treatment for early-stage cervical cancer, most patients receive radiotherapy, either as an adjuvant treatment after surgery or as the primary treatment for locoregionally advanced disease. Improved long-term survival has led to an increase in the proportion of women exposed to the late adverse effects of radiotherapy. As in prostate cancer, there is considerable variability in the reported incidence of these late adverse effects [32,33,34,35]. Since cervical cancer presents at a relatively young age (i.e., with a peak incidence between 35 and 45 years), during a phase of life with many family and work responsibilities, these adverse effects can significantly affect QoL after treatment [36,37,38].

Personalized treatment strategies that account for individual risk factors could potentially mitigate the development of severe adverse effects following pelvic radiotherapy, thereby enhancing post-treatment QoL. However, the current radiotherapy protocols predominantly follow standardized guidelines, prescribing radiation doses, fractionation schedules, and target volumes based on the average patient’s tolerance. This one-size-fits-all approach fails to account for the diverse physiological and genetic variations among individuals. With a growing population of long-term cancer survivors, the adoption of personalized treatment strategies could yield substantial benefits for numerous patients. Despite extensive research into both treatment-related and patient-related factors influencing late radiation toxicity, the development of a robust predictive model remains a challenge. Comprehensive and renowned reviews are available on radiation dose–volume effects [39,40,41,42,43]. With the development of more conformal techniques that reduce the radiation doses to healthy tissue, there is an increasing need to understand patient-specific determinants of late toxicity better. This narrative review aims to critically examine various scoring methods for late radiation toxicity and to explore the key patient-related risk factors implicated in the onset of late adverse effects. A narrative methodology was chosen to provide a broad and comprehensive perspective on this multifaceted topic. To ensure clarity and manageability, this review specifically addresses prostate and cervical carcinomas, which represent two distinct treatment approaches with well-established radiotherapy protocols and clinically significant long-term toxicity profiles. The terms guiding our search in the PubMed database included “prostate cancer”, “cervical cancer”, “gynecological cancer”, “radiotherapy” or “external beam radiotherapy”, “late toxicity”, “scoring”, “quality of life”, “genetic predisposition”, “biomarkers”, “clinical risk factors”, “predictive factors”, “predictive assay”, “deep learning”, and “personalized treatment”. Relevant articles were identified using these search terms, and the reference lists of the selected articles were reviewed to identify additional relevant studies.

## 2. Part I: Scoring of Late Radiation Toxicity

Reliable quantification of late radiation toxicity is crucial for advancing cancer care. While tumor outcome reporting is fairly standardized, there is no general consensus on the best method for quantifying treatment toxicity [3,7,44]. Several systems are available for physicians to grade the severity of toxicity, and these systems are based on symptoms and interventions [9,10,45,46,47,48]. Radiation toxicity may also be assessed using patient-administered questionnaires, known as Patient-Reported Outcome Measures (PROMs). Due to the variety of the assessment methods, the late adverse effects reported in the literature are not easily comparable [9,49,50,51]. Furthermore, the completeness of the toxicity information reported varies widely due to differences in condensing and summarizing late toxicity data [7]. Some trials report all levels of toxicity, while others only present the most severe cases. Information is often aggregated by site or grade, resulting in a further loss of detail. Generally, the recurrence or duration of late toxicity is not addressed, with the crude incidence (the worst grade during follow-up) frequently used as the sole statistical method for summarizing toxicity [7]. In this section, we will address several commonly used scoring instruments and discuss their pros and cons. Further insights into the challenges of reporting are beyond the scope of this review.

### 2.1. Physician-Reported Scoring of Adverse Events

Quantifying and evaluating late effects have been critical concerns for radiation oncologists for decades. In the late 1970s, the Late Radiation Morbidity Scoring Criteria were developed through a collaborative effort between the Radiation Therapy Oncology Group (RTOG) and the European Organisation for Research and Treatment of Cancer (EORTC) [10]. With the RTOG/EORTC Late Radiation Morbidity Scoring Scheme, a grading system was introduced that categorizes the severity of radiation effects on a scale from 0 to 5. A score of 0 indicates the absence of radiation toxicity, while grades 1 through 3 represent a progression from mild to moderate and severe effects, respectively. More critical conditions such as necrosis, perforations, and fistulas are classified as grade 4. When radiation effects lead to mortality, grade 5 is assigned. The RTOG started to use these criteria to report toxicity in patients enrolled in all studies from 1981 onward [10].

In the early 90s, the EORTC and RTOG working groups again joined forces to update their systems for assessing late toxicity [9,45,46]. This effort led to the introduction of the LENT-SOMA system, which stands for Late Effects Normal Tissues—Subjective, Objective, Management, and Analytic. The LENT-SOMA scales, published in 1995, were seen as a logical extension of the previous scales [9,45,46]. They address both objective and subjective toxicity, integrating clinical test results and medical interventions. Despite its thoroughness and detailed approach, the LENT-SOMA system has not been adopted widely due to its extensive format and the challenges it poses for routine application.

In current daily practice and research, physicians commonly grade late radiation toxicity using the Common Terminology Criteria for Adverse Events (CTCAE) [7,47]. Version 3.0 of the CTCAE (CTCAEv3.0), published in 2003 by the National Cancer Institute (NCI), represented the first comprehensive, multimodality grading system for reporting both the acute and late effects of cancer treatment [47,52]. It was developed through a series of meetings, with the fourth meeting, the Late Effects Workshop, involving a diverse group of multidisciplinary experts. The CTCAEv3.0 includes approximately 900 site-specific definitions for adverse events (AEs), greatly enhancing the standardization of AE reporting. This standardization facilitates outcome comparisons across clinical trials and institutions while promoting comprehensive recognition and documentation of adverse effects. The CTCAE was revised in 2009 and 2017, resulting in the current version, CTCAEv5.0 [48]. Although the CTCAE was developed and updated through a consensus-based process, some content validity is conferred through more than 40 years of relatively widespread use and iterative improvements.

Similarly to other grading systems, the CTCAE classifies AE severity into 5 grades: from asymptomatic or mild symptoms (grade 1) to death related to an AE (grade 5). Toxicities are graded based on specific criteria related to the severity of symptoms, clinical signs, and laboratory results. However, because the symptom interpretation can vary among physicians, grading toxicities can sometimes be subjective [51,53]. One physician might initiate treatment earlier than another, resulting in a relative “upgrading” of severity. This interobserver variability indicates that a perfect agreement between physician and patient assessments is unlikely. Studies have shown a tendency for healthcare professionals to underestimate symptoms in terms of both their frequency and severity [54,55,56,57,58,59,60,61]. Additionally, AE reporting by staff typically occurs at clinic visits, so symptoms that arise between visits may be missed. Despite these limitations, numerous studies have established significant correlations between CTCAE grades and objective clinical parameters [27,28,50,56]. Regrettably, the system has not yet been universally adopted in routine clinical practice.

### 2.2. Patient-Reported Outcome Measures

Patient-Reported Outcome Measures (PROMs) are tools used to assess health status or health-related quality of life (HRQoL) from a patient’s perspective. PROMs are valuable because they provide data directly from patients, without their interpretation by physicians or others. These data can include information on symptoms, functional status, psychological well-being, and overall HRQoL. The EORTC quality of life questionnaire (QLQ) is one of the most widely used questionnaires in Europe for cancer patients and is extensively used worldwide [62,63]. The latest version of the core questionnaire, the QLQ-C30 version 3.0, is the result of over two decades of collaborative research and contains 30 items in 9 subcategories, including 5 functional scales, a global health status/QoL scale, 3 symptom scales, and 6 single items [16,63]. Additionally, in the EORTC, disease-specific modules have been developed, including for prostate cancer (EORTC QLQ-PR25) and cervical cancer (EORTC QLQ-CX24) [64,65]. These modules can be used independently or as supplementary modules to the EORTC QLQ-C30 to assess aspects of HRQoL specific to men with prostate cancer and women with cervical cancer.

Historically, PROMs have primarily been used in clinical trials to evaluate the treatment efficacy and HRQoL across different treatment groups [62,66]. For this purpose, these measures have undergone rigorous validation [66,67]. Over time, their application has been broadened to include individual patient management and monitoring in clinical practice [62,68,69]. In addition, there is increasing interest in using PROMs to assess the incidence and severity of symptomatic adverse events.

### 2.3. Inclusion of One, the Other, or Both Perspectives

The inclusion of patient-reported or clinician-reported symptomatic AEs in clinical trials and drug labels, or a combination of both, has been a topic of debate for over a decade [70,71]. Direct patient reporting of adverse symptoms has been proposed as an efficient alternative, potentially reducing data loss, misinterpretation, and transformation [70,72,73]. However, clinician reporting of adverse symptoms is unlikely to be discontinued, as it is well established and generally more complete than patient-reported data [15,56,74]. Clinician-reported CTCAE assessments have shown strong correlations with clinical endpoints such as hospitalization and survival, whereas patient-reported outcomes (PROs) provide a more accurate reflection of daily health status [56]. Notably, PROs may improve over time as patients psychologically adapt to new life circumstances, a phenomenon known as a response shift [75]. Since both perspectives offer clinically meaningful information, incorporating both types of data seems warranted. A suitable approach could involve health professionals continuing to track objective toxic effects while patients self-report their symptoms, followed by clinician reviews and annotation [76,77]. Integrating patient-reported outcomes into AE reporting could enhance the accuracy and efficiency of subjective AE data collection. Currently, the PRO-CTCAE appears to be the most suitable PROM, as it was specifically designed to complement the CTCAE in evaluating toxicity [78]. Furthermore, it has been validated to ensure its reliability, validity, and usefulness in a clinical trial setting [79,80,81].

## 3. Part II: Clinical Risk Factors

Numerous clinical factors have been identified as potential risk factors for late toxicity after pelvic radiation therapy, including age, BMI, smoking behavior, comorbidities such as diabetes mellitus and inflammatory bowel disease, previous abdominal surgery, pretreatment symptoms, and severe acute toxicity [4,6,23,24,28,34,82,83,84,85,86,87,88,89,90,91,92,93,94,95,96,97,98,99]. In men with prostate cancer, prior TURP and hormonal treatment have been implicated as possible risk factors [6]. For most of these proposed risk factors, however, consistent evidence from independent and prospective studies remains lacking.

One such example is a study from the Dutch dose escalation trial, where Peeters et al. found that diabetes mellitus was associated with an increased risk of developing two specific complications: “needing incontinence pads for rectal discharge” and “nocturia (≥4)” [6]. In this trial, 669 prostate cancer patients were randomized to receive radiotherapy at doses of either 68 Gy or 78 Gy. Late toxicity was prospectively assessed using slightly adapted RTOG/EORTC scales and 12 specific complications. However, in a subsequent study from the same trial, with the follow-up period extended from 31 to 44 months and a focus on gastrointestinal toxicity, the association between diabetes and toxicity could not be confirmed [93]. Jensen et al. specifically examined patient- and treatment-related risk factors associated with the incidence and persistence of late diarrhea after image-guided adaptive brachytherapy in women with locally advanced cervical cancer (LACC) [90]. In this prospective study, diabetes had a consistent (i.e., measured using both CTCAE and EORTC endpoints) and strong impact on the persistence of late diarrhea (HR of up to 3.23). The seemingly increased risk in diabetic patients could be explained by elevated baseline levels of inflammatory cytokines and reactive oxygen species (ROS), which contribute to chronic oxidative stress, leading to endothelial dysfunction and subsequent microvascular damage [92,100]. Radiotherapy further exacerbates these processes by inducing additional ROS and promoting inflammatory responses [100,101]. The combined effect of these interconnected mechanisms likely results in a synergistic interaction that amplifies late radiation-induced toxicities. Interestingly, in a study involving 515 women with LACC by Laan et al., diabetes was not linked to severe late bowel toxicity [34]. Instead, this study found that smoking, severe acute bowel toxicity, major abdominal surgery, hypertension, low socioeconomic status, and low BMI were significant factors contributing to severe late bowel toxicity.

There is a reliable body of evidence showing that prior TURP, smoking, acute toxicity, and previous abdominal surgery are associated with an increased risk of late toxicity. Given the substantial evidence linking prior TURP to late urinary toxicity, most centers avoid planning EBRT immediately after TURP [6,28,82,94,95,102]. Peeters et al. identified “urinary obstruction” and “dysuria requiring drugs” as the most prominent indicators of late genitourinary toxicity in patients with prior TURP [6]. These findings are consistent with those from a large prospective study by Sandhu et al., which reported a significantly higher risk of urethral stricture requiring dilatation in patients with prior TURP compared to those without [95].

Smoking is also recognized as a serious risk factor for toxicity [6,34,86,87,89,90]. Eifel et al. reviewed the records of nearly 3500 patients who underwent radiotherapy for FIGO stage I or II cervical cancer, assessing the patient characteristics, treatment details, and outcomes [87]. Ninety-nine percent of patients were followed for at least 3 years or until death. Heavy smoking (≥1 packs/day) emerged as the strongest independent predictor of major complications. Smoking was particularly associated with small bowel complications, with women who smoked less than one pack per day also experiencing a significantly increased risk. The risk was dose-related, with the actuarial rate of major small bowel complications at 10 years increasing more than five-fold in the heaviest smokers (11.1%) compared to those who had never smoked (2.1%). Interestingly, this study also identified a BMI below 22 as a significant risk factor for rectal and small bowel complications, possibly due to reduced physical protection from radiation exposure due to less mesenteric fat, as well as lower nutritional reserves, which could impair tissue repair after radiation damage [99,103,104,105,106,107]. A more recent prospective study linked smoking to an increased risk of CTCAE grade ≥3 and grade ≥2 late diarrhea, with hazard ratios of 3.02 and 1.47, respectively [90]. In addition, the continuation of smoking during treatment not only increases the risk of severe acute and late toxicity but also leads to poorer oncological outcomes [108,109,110,111]. Therefore, it is essential for oncological caregivers to address smoking behavior, discuss cessation strategies, and, if desired, refer patients for smoking cessation support [112,113,114].

Many studies have shown an association between early and late toxicity [23,24,34,82,83,93,94,95,96,97]. Apart from a direct dose effect, several studies have demonstrated a relationship between acute and late toxicity that is independent of dose [23,83,96,97,98]. This phenomenon, known as the consequential late effect, occurs when the acute radiation response leads to tissue damage that eventually manifests as late effects after a latent, symptom-free interval. In an observational multicenter prospective study involving over 20 Italian institutes (AIROPROS 0102), late fecal incontinence appeared to mainly be a consequential effect after acute severe incontinence. Acute incontinence emerged as the most predictive factor for both actuarial late incontinence and chronic late incontinence, with odds ratios of 6.9 and 4.34, respectively [97]. This finding was confirmed by another AIROPROS 0102 study [96].

Additionally, AIROPROS 0102 provided convincing evidence of previous abdominal surgery being a risk factor for late toxicity [96]. It was found that once appropriate dose–volume constraints are applied, surgery is one of the major predictors of late rectal toxicity. Among the various types of surgery, cholecystectomy and appendectomy were particularly significant, especially in relation to severe late bleeding, with odds ratios of around 6 [96]. Similarly, a study from a Dutch dose escalation trial in prostate cancer also identified previous abdominal surgery as a key prognostic factor for late gastrointestinal toxicity [6]. This factor was found to be more important than the studied dose levels of 68 Gy and 78 Gy. Consistent with the findings from AIROPROS 0102, patients with a history of surgery were particularly prone to developing late rectal bleeding. The role of previous abdominal surgery as an independent prognostic factor for gastrointestinal toxicity was further confirmed in a subsequent study from the same trial, which incorporated dosimetric parameters into the multivariate analysis [93]. An elevated risk of severe late (small) bowel toxicity was also observed in women treated for cervical cancer who had undergone previous abdominal surgery [4,34,85].

As noted, there are insufficient data on the degree of risk posed by most other factors, including age (both younger and older), BMI (i.e., low and very high), race (i.e., being black), hypertension, low socioeconomic status, diabetes mellitus, collagen vascular disease, inflammatory bowel disease, and physical inactivity. This is likely due to several factors: the wide variety of methods used to evaluate toxicity, differences in the primary outcomes reported (such as 5-year actuarial versus 10-year incidence or overall major complications versus grade ≥2 diarrhea), variations in patient populations, differences in the statistical methods and handling of confounding variables (with the dose–volume parameters of the organs at risk not consistently being included in multivariable analyses), the retrospective nature of many studies, short follow-up periods, and small sample sizes. Together, these limitations contribute to the conflicting evidence on the patient-related risk factors for late toxicity after pelvic irradiation. Unsurprisingly, many studies conclude by emphasizing the need for validation of their findings in larger, prospective cohorts with longer follow-up periods. Table 1 presents several clinical risk factors for late radiation toxicity following EBRT, as determined in men with prostate cancer and women with cervical cancer.

Only factors with (borderline) significant risks are shown. Grading scales are provided when they can be expressed in a single word; otherwise, they are detailed in Supplementary Table S1, along with the treatment and study information and whether dosimetric parameters were included in the multivariate analysis.

## 4. Part III: Genetic Risk Factors

Over the past few decades, extensive research has been conducted on the role of genetic variation in the cellular and clinical radiosensitivity of normal tissues. Most of these studies have focused on genes involved in DNA damage recognition and repair, free radical production and scavenging, and inflammatory responses. Despite these efforts, the genetic determinants of late radiation toxicity remain poorly understood, and reliable predictive markers have yet to be identified. This section provides an overview of genes that may be relevant to late radiation toxicity, as well as promising predictive assays for normal tissue radiation sensitivity.

### 4.1. Observations from Patients with Inherited Disorders

The hypersensitivity to radiation therapy observed in certain rare autosomal recessive genetic disorders has highlighted the importance of specific genes. For example, the death of an 11-year-old boy from radiation toxicity was linked to mutations in both copies of the ATM gene, which causes ataxia telangiectasia [116]. This unfortunate event prompted further investigation into the role of the ATM gene in DNA damage repair. The ATM gene encodes a serine/threonine protein kinase that phosphorylates proteins involved in cell-cycle checkpoint regulation, DNA repair mechanisms, and apoptosis in response to DNA double-strand breaks (DSBs) [117,118]. Mutations in the ATM gene disrupt these critical damage signaling pathways, resulting in increased radiosensitivity and cancer predisposition, among other health issues [119,120,121,122,123].

Increased cellular radiosensitivity has also been documented in several inherited diseases, some of which share phenotypic similarities. These include ataxia-telangiectasia-like disorder (an MRE11 gene defect), radiosensitive severe combined immunodeficiency (a DCLRE1C or PRKDC gene defect), Nijmegen breakage syndrome (an NBS1 gene defect), Nijmegen breakage syndrome-like disorder (a RAD50 mutation), RIDDLE syndrome (an RNF168 mutation), Cornelia de Lange syndrome (SMCL1A and SMC3 mutations), and LIG4 syndrome (a DNA Ligase IV gene defect). These disorders are all caused by mutations in genes that are crucial for the recognition and repair of damaged DNA [124,125].

Several types of DNA damage can occur following exposure to ionizing radiation, with DNA DSBs being the most severe [126]. When DNA DSBs are induced, specific repair proteins bind to or near the site of the break, initiating the recruitment of additional proteins that form a complex to facilitate repair. The gene products of MRE11, RAD50, and NBS1 assemble into the MRN complex, which plays a critical role in the initial processing of DNA DSBs prior to repair. In addition, the MRN complex is essential for ATM activation in response to damage [127,128]. ATM activity is central to the detection of DNA DSBs and their subsequent repair, the latter primarily through homologous recombination (HR) or non-homologous end joining (NHEJ) [129,130,131]. In summary, the gene products of MRE11, NBS1, and ATM interact within a common cellular pathway essential for the effective recognition and repair of DNA DSBs.

Mutations in single genes may have clinical implications only if they result in significantly reduced activity of a gene product that is critical within a common cellular pathway. In addition to increased cellular radiosensitivity, patients with ataxia telangiectasia, LIG4 syndrome, and Nijmegen breakage syndrome also exhibit clinical radiosensitivity. The single gene mutations in these rare autosomal recessive disorders evidently have a strong effect on the activity of the gene products. However, a mutation in only one copy of a critical gene does not necessarily result in an increased risk of radiation-induced toxicity. For example, although experiments with fibroblasts and lymphocytes from ATM heterozygotes have demonstrated increased cellular radiosensitivity, the literature remains inconclusive regarding the association between ATM heterozygosity and clinical radiosensitivity [123,125,132,133,134,135]. Furthermore, heterozygous carriers of other syndromes do not exhibit an increased risk of radiation toxicity. Comprehensive overviews of inherited syndromes and genes associated with clinical and/or cellular radiosensitivity are provided by West and Barnett and Bergom et al. [124,125].

### 4.2. Candidate Gene Association Studies

Marked differences in clinical radiosensitivity cannot be attributed solely to known genetic defects and their associated syndromes, as these conditions are rare, whereas severe radiotoxicity occurs in 5–10% of patients. Therefore, it has been hypothesized that more subtle genetic variations, such as single-nucleotide polymorphisms (SNPs), may contribute to individual differences in normal tissue radiosensitivity. In predominantly retrospective studies of prostate cancer cohorts, SNPs in candidate genes encoding proteins involved in DNA damage recognition and repair, inflammation, and steroid metabolism have shown potential for predicting the individual risk of late radiation toxicity [136,137,138,139,140,141]. For example, variations in genes such as ATM, SOD2, XRCC1, XRCC3, MLH1, LIG4, ERCC2, and CYP2D6 have been associated with an increased risk of adverse reactions to radiotherapy (Table 2). In a study of 48 prostate cancer patients, Cintra et al. found an association between two TP53 polymorphisms and radiation-induced toxicity. One polymorphism was linked to acute skin toxicity, while the other was associated with chronic urinary toxicity. No such associations were found for ATM or MDM2 polymorphisms [141]. Interestingly, individuals with autosomal dominant syndromes that confer an increased risk of radiation-induced second cancers, such as those heterozygous for pathogenic germline mutations in the TP53 tumor suppressor gene (Li–Fraumeni syndrome), do not exhibit an increased risk of radiation toxicity [125].

For gynecological cancers, the available data are considerably more limited. In a study of 62 patients with cervical or endometrial carcinomas, de Ruyck et al. examined genetic variants of XRCC1, XRCC3, and OGG1. They found that a specific variant of XRCC3 was associated with an increased risk of grade 2 or higher late radiation toxicity. In contrast, carriers of an XRCC1 polymorphism showed a significantly reduced risk of late toxicity (Table 2) [142].

Candidate gene studies, such as those described above, have yielded many positive results; however, most of these findings have been inconsistent and difficult to replicate [144,145,146]. This perspective is supported by an independent validation study by Barnett et al., which involved 1613 patients and found no significant associations between 92 candidate SNPs and radiation toxicity [144]. Nevertheless, several studies have consistently supported an association for one common SNP in the ATM gene (rs1801516; Asp1853Asn, G > A). A comprehensive meta-analysis of individual patient data, including 5456 patients with breast or prostate cancer, found a modest but statistically significant increase in the risk of radiation toxicity among carriers of the Asn allele. The odds ratios were approximately 1.5 for acute toxicity and 1.2 for late toxicity [147].

### 4.3. Non-Candidate Gene Association Studies

Genome-wide association studies (GWASs) have been initiated to provide more definitive insights into the role of SNPs in (predicting) radiation toxicity. In contrast to candidate studies, GWASs involve genotyping patients for a large number of SNPs that may or may not be common. The first study to use a genome-wide approach reported a significant association between an SNP (rs2268363) in the follicle-stimulating hormone receptor (FSHR) gene and the development of erectile dysfunction after radiotherapy for prostate cancer. In this study, the DNA of 79 African American men was screened for approximately 909,000 SNPs, which is a typical number for GWASs. Carriers of the minor allele were seven times more likely to develop erectile dysfunction after EBRT than those who did not possess this SNP [148]. More recent publications by the same author did not confirm this association, which was attributed to the different ethnic composition of the cohorts studied [149].

Due to the large number of hypotheses being tested simultaneously, adequately powered GWASs typically require thousands of patients. This need underscores the importance of international collaboration and data sharing, which led to the establishment of the Radiogenomics Consortium (RGC) in 2009 [150].

A GWAS of 741 prostate cancer patients from the RADIOGEN cohort identified an association between SNPs within the TANC1 locus (2q24.1) and the development of overall late toxicity [151]. When two replication cohorts (n = 849) were included, the meta-analysis *p*-value for a specific SNP (rs7582141) decreased to 4.64 × 10^−11^, well below the threshold for genome-wide significance (typically set at 5 × 10^−8^). An individual carrying one copy of the risk allele was estimated to have a sixfold increased risk of developing late toxicity compared with a non-carrier. In a GWAS by Barnett et al., several associations between SNPs and the risk of toxicity were identified in breast (n = 1194) and prostate (n = 579) cancer patients [152]. However, these significant associations could not be validated at the genome-wide significance level in the replication cohorts. This study was powered to detect SNPs with tumor-site-independent effects but found stronger associations with tumor-site-specific toxicity. This may indicate that not all genetic variants have a universal effect on clinical radiosensitivity. Both studies used a multi-stage approach, starting with a small first-stage cohort that was analyzed for a genome-wide panel of SNPs. Only the most significant SNPs were then genotyped in the validation cohorts. This approach may have resulted in missing true positive SNPs, as they were not tested across the entire datasets.

To maximize the statistical power in identifying additional risk variants with smaller effect sizes, the RGC conducted a meta-analysis of four GWASs in 2016 [145]. These studies—RAPPER, RADIOGEN, Gene-PARE, and CCI-EBRT—focused on prostate cancer patients, 1565 in total, who received curative radiotherapy. The meta-analysis reported two SNPs that reached genome-wide significance: rs17599026 (5q32.2), associated with urinary frequency (OR = 3.12), and rs7720298 (5p15.2), associated with a decreased urinary stream (OR = 2.71). These SNPs are located in the intronic regions of genes (KDM3B and DNAH5, respectively) that are expressed in tissues adversely affected by pelvic radiotherapy. This study did not confirm the associations with toxicity for any previously studied candidate gene SNPs, nor did it replicate the SNPs discovered in earlier GWASs [145]. This may be because most of the common variants identified by GWASs have more modest effects (ORs = 1.15 to 1.5) than this study was powered to detect. As a result, the RGC undertook a subsequent meta-analysis of six GWASs that included 3871 prostate cancer patients [153]. This study confirmed the previous associations of KDM3B (OR = 1.23) and DNAH5 (OR = 1.37), as well as that of ATM (rs1801516; OR = 1.37), with overall toxicity, although the effect sizes were attenuated. The association of TANC1 with overall toxicity was not confirmed (OR = 0.98). In addition, the meta-analysis identified four new genomic regions associated with radiotoxicity. SNP rs17055178 on chromosome 5 was linked to rectal bleeding, SNP rs10969913 on chromosome 9 was associated with a decreased urinary stream, and SNPs rs11122573 and rs147121532 on chromosome 1 were linked to hematuria. These new loci appear to be involved in gene regulation rather than in coding sequences.

### 4.4. Predictive Assays Based on the Cellular Functional Response to Ex Vivo Irradiation

Early efforts to develop predictive assays for clinical radiotoxicity stemmed from the observation that cells derived from patients with ataxia telangiectasia were more radiosensitive ex vivo than those from healthy controls [154,155]. The initial assays used patient-derived skin fibroblasts or lymphocytes to measure the radiation-induced cell death through clonogenic assays, with metrics such as the surviving cell fraction after 2 Gy correlating with clinical radiotoxicity [156]. Although initially promising, these assays were labor-intensive, had poor technical validity, and showed a low predictive value in subsequent validation studies. This led to the exploration of alternative assays using different cell types and methods. One such example is the radiation-induced lymphocyte apoptosis (RILA) assay, which was developed by Ozsahin et al. in the 1990s and has since been optimized [157]. In this assay, lymphocytes are irradiated ex vivo at a high dose rate, and the resulting level of apoptosis is readily detected using flow cytometry, which can then be correlated with clinical radiotoxicity. Over the years, the RILA assay has been prospectively validated in multicenter cohorts, consistently demonstrating an inverse correlation between the RILA scores and late toxicity across various cancer types [158,159,160]. For example, in a prospective multicenter trial in 383 prostate cancer patients, a RILA score exceeding 15% was associated with lower grade ≥2 genitourinary and gastrointestinal toxicities [160]. Similarly, in a smaller prospective study of cervical cancer patients, higher RILA scores were significantly linked to lower levels of sexual, rectal, and urinary toxicity [161]. In addition, the RILA assay has emerged as one of the most robust and reproducible tests for assessing radiation-induced toxicity [162].

Although radiation can cause various types of DNA damage, the induction of double-strand DNA breaks is fundamental to both the therapeutic effects (i.e., tumor cell death) and adverse effects (i.e., damage to normal cells) of radiotherapy. Several functional assays have been developed to measure different aspects of DNA DSB recognition and repair in patient-derived cells. Among the most promising of these functional assays is the y-H2AX foci analysis. In response to DNA DSBs, nuclear ATM phosphorylates the H2AX histones at the break sites, producing y-H2AX foci. This phosphorylation is one of the earliest steps in the response of mammalian cells to radiation-induced DNA damage [163,164]. The kinetics of y-H2AX foci—specifically their formation and resolution—can be observed over time using immunofluorescence detection, allowing for the quantification of DNA DSB induction and repair. There is a known difference in the clinical radiosensitivity between patients with ataxia telangiectasia and healthy controls. In addition, Kato et al. found that y-H2AX foci analyses can distinguish between cell samples from individuals with normal ATM function, ATM heterozygotes, and affected ATM probands [123]. This finding demonstrates the potential of functional assays to identify the genetic factors that influence individual radiosensitivity, which may be useful in predicting adverse reactions to radiotherapy. Following this discovery, several research groups have shown that the number of residual foci (typically determined 24 h after irradiation) is significantly higher, and the decay of foci is significantly lower, in irradiated cells from patients with radiation toxicity [27,28,165,166,167,168]. However, other studies have found no evidence of an association between the y-H2AX foci kinetics and radiation toxicity [74,169,170]. Possible explanations for these discrepancies include small sample sizes, retrospective study designs, and the lack of detailed dosimetry. Moreover, this assay has not been standardized across laboratories, raising concerns about its technical validity. For example, one of the studies with negative results used a flow cytometric assay, which is known to have a lower sensitivity compared with visual assessment of the foci using manual or automated microscopy. Comprehensive studies with large prospective cohorts are needed to validate the suitability of y-H2AX foci analysis in clinical settings further.

### 4.5. Predictive Assays Based on Polygenic Risk Scores Using Artificial Intelligence

The polygenic basis of clinical radiation toxicity is increasingly recognized. Given the small effect sizes and low penetrance of most SNPs, it is unlikely that any individual SNP exerts a clinically relevant effect or can serve as a reliable marker of radiation toxicity. Consequently, recent research has shifted toward multi-SNP modeling with the goal of developing polygenic risk scores (PRSs).

Machine-learning-based multivariate modeling offers a promising approach by simultaneously considering numerous relevant SNPs and combining their small effects to achieve a greater predictive power. By aggregating the effect sizes of these predictors, a more reliable predictive model can be constructed. Both Oh et al. and Lee et al. used a preconditioned random forest regression method on GWAS cohorts of 368 and 324 prostate cancer patients, respectively [171,172]. Oh et al. found that this method resulted in a better predictive performance than that of the existing computational methods in predicting the risk of rectal bleeding and erectile dysfunction [171]. Similarly, Lee et al. applied this method to predicting four urinary symptoms and achieved a significant area under the curve (AUC) of 0.70 for a weak stream on the hold-out validation data, outperforming competing methods [172]. Efforts are underway to validate these and other predictive models and biomarkers.

One notable effort is REQUITE, an international prospective cohort study of patients undergoing radiotherapy for breast, lung, or prostate cancer [60]. As one of the largest studies of its kind, REQUITE collected blood samples and standardized longitudinal data from over 4400 patients, aiming to establish a resource for the multinational validation of models and biomarkers that predict the risk of late radiation toxicity. Imputed genotype data are available for 4223 patients, and RILA assay data are available for a subset of 1319 patients.

Massi et al. used a deep learning algorithm to analyze data from 1401 prostate cancer patients enrolled in the REQUITE study. Of the 43 SNPs previously reported in the literature to be associated with five specific toxicity endpoints, 24 were validated as markers for identifying patients with toxicity [173]. Building on these findings, Franco et al. investigated the potential added value of incorporating SNP-SNP interactions into PRSs. These interactions account for the combined influence of multiple SNPs on the phenotype. In the context of radiation sensitivity, one SNP might increase the risk of toxicity, while another might reduce it. The presence of both SNPs together could result in an intermediate level of risk or even a completely different risk profile than that with either SNP alone. Franco et al. focused on the top 30% most relevant SNPs among the 43 initially studied by Massi et al. for each of the five toxicity endpoints [174]. They found that incorporating SNP-SNP interaction effects improved the ability to discriminate patients with toxicity compared to that using classical summation (i.e., the additive contributions of individual SNPs). For example, the AUC for late urinary frequency increased from 0.60 to 0.78. This method, which accounts for SNP-SNP interactions rather than simple additive effects, is expected to improve the integration of genetic information into normal tissue complication probability (NTCP) models. However, both the methods used by Massi et al. and Franco et al. require further evaluation in independent studies to determine the most effective PRS for inclusion in integrated NTCP models.

## 5. Discussion on the Future Directions and Clinical Integration of Predictive Models for Radiation Toxicity

A predictive assay for late radiation toxicity would enable more personalized treatment planning, minimizing the toxicity to the more sensitive minority while maximizing the therapeutic ratio for the majority. As radiotherapy becomes increasingly conformal, reducing the doses to the healthy tissue, understanding the patient-specific determinants of toxicity is crucial.

The strongest evidence for clinical factors associated with toxicity includes smoking behavior, acute toxicity, previous abdominal surgery, and, in men, prior TURP. For most other factors, the evidence remains conflicting or weaker. In the genetics of radiosensitivity, many SNPs have been identified through candidate gene studies and GWASs, but only a few have been reliably replicated. In recent decades, the polygenic basis of radiation toxicity has been increasingly recognized, and AI has emerged as a powerful tool for extracting signals from large datasets. The current research has shifted toward multi-SNP modeling to develop polygenic risk scores (PRSs). In addition to advancements in AI, predictive assays based on cellular functional responses to ex vivo irradiation continue to be explored. Renewed interest in these assays has been sparked by improvements in their reproducibility and technical feasibility, with the RILA assay showing the most promise.

A commercially available RILA assay, NovaGray, has been developed for breast and prostate cancer. The ability of the NovaGray RILA breast test to predict radiation-induced breast fibrosis is currently under investigation (NCT04342546). As the risk thresholds are thought to differ between cancer types, clinical validation for each type may be required. PRSs are still largely in the development phase. Originally, it was assumed that certain genetic variations would be associated with radiation toxicity across all tissues, regardless of the tumor location. This assumption was based on our understanding of rare genetic variants in DNA damage response genes, which significantly influence the radiosensitivity and toxicity risk across different tissues [175]. However, Barnett et al. found that genetic associations with radiation toxicity tend to be specific to particular tumor sites, which aligns with clinical studies showing a lack of consistent toxicity across multiple endpoints within individual patients [152]. Consequently, PRSs are likely to be specific to the type of normal tissue involved, making independent clinical validation in different cancer types essential. In these validation studies, non-genetic risk factors—such as radiation dose, irradiated volume, and comorbidities—must also be quantified. Although many studies have focused solely on genetic markers, accounting for these non-genetic factors is crucial to reliably assess the impact of genetics.

Further research is needed to determine how these predictive tests can be reliably implemented in clinical practice. While PRSs for normal tissue toxicity are likely to be tissue-specific, some underlying genetic mechanisms may still have broader relevance. Specifically, genetic factors that influence the radiosensitivity of normal tissues may also influence the radiosensitivity of tumors. It is crucial to investigate whether toxicity biomarkers can also serve as predictors of tumor radiosensitivity and guide treatment decisions. Some studies have suggested an association between radiation toxicity and cancer outcomes [176,177,178]. For example, Eade et al. found that prostate cancer patients who experienced acute epithelial radiation toxicity had a significantly longer freedom from biochemical failure [178]. While these findings are promising, they have not yet been fully established. A key direction for future research could be the use of paired testing to identify genetic biomarkers that predict both radiation toxicity in normal tissue and tumor response to radiotherapy. Such an approach would allow researchers to determine whether the same genetic factors influence both outcomes, which could help guide personalized radiotherapy strategies in clinical practice. By analyzing both somatic mutations in tumor tissue and germline mutations in normal tissue, it is possible to identify the shared genetic basis of these responses.

In addition, more research is needed to determine exactly how treatment should be guided based on an individual patient’s risk profile. For example, a prostate cancer patient with a predictive assay indicating radiosensitivity could opt for surgery or active surveillance if his PSA and Gleason scores supported those choices. If not, the assay could guide the treatment planning for radiotherapy. However, the precise strategies for tailoring treatment to optimize outcomes remain unclear. A high-risk patient might benefit from an alternative plan with daily image guidance and reduced margins to avoid critical organs at risk better, thereby reducing the predicted toxicity. Additional interventions, such as rectal spacers, could also be considered, but the question remains as to when and at what level of risk these should be implemented. Furthermore, if patients prone to normal tissue damage do indeed have more radiosensitive tumors, they may be able to be cured with lower radiation doses. But what should the adjusted dose be? On the other hand, for patients predicted to be at low risk, dose escalation may improve tumor control while maintaining a low risk of normal tissue toxicity—but by how much should the dose be increased? In conclusion, while there are many ideas for personalizing treatment and significant interest among radiation oncologists in ordering predictive tests, there is a lack of consensus guidelines on how genetic testing should inform the treatment decisions for cancer patients undergoing radiotherapy [179]. To address this, future trials designed to evaluate genomically guided radiotherapy should compare patients receiving genomically adjusted doses—taking into account both normal tissue toxicity susceptibility and tumor radiosensitivity—with those receiving the standard protocols. Such trials would provide critical evidence on how to personalize the radiation doses based on genetic profiles and optimize outcomes for cancer patients undergoing radiotherapy.

While the potential for personalized treatment based on an individual patient’s risk profile is clear, the path forward requires careful consideration of how best to integrate these insights into clinical practice. As we look to the future, the most effective strategy will likely involve combining one or more biomarker assays with radiation dosimetry and clinical risk factors to generate a personalized risk score during the treatment planning phase. It is expected that some of the predictive assays for toxicity discussed in this review will soon be integrated into clinical practice, improving the ability of radiotherapy to treat cancer while minimizing damage to healthy tissues.

## Figures and Tables

**Table 1:** Patient factors associated with late radiation toxicity in cervical and prostate cancer patients.

Factor	Complication Analysis and Risk	Ref.
	Cervical Cancer	
Age >70 years	Higher risk (incidence: 44% vs. 26%) of rectal sequelae (G1–3; Esche et al. [115]) in patients >70 years vs. patients ≤70 years. MV-LRA: RR = 2.2, *p* = 0.020.	Chen (2000) [84]
Age <40 years	Higher risk (3-year actuarial rate: 16% vs. 9%) of major complications in patients <40 years vs. patients ≥40 years. Univariate analysis using the Mantel–Haenszel test: *p* = 0.0046. MV-CRA: RR = 1.60, *p* = 0.0644.	Lanciano (1992) [85]
Advanced stage	Higher risk (incidence: 36% vs. 10%) of rectal sequelae (G1–3; Esche et al. [115]) in patients with stage IIb-IVa disease vs. patients with stage Ib-IIa disease. MV-LRA: RR = 3.6, *p* = 0.030.	Chen (2000) [84]
	Higher risk (5-year actuarial risk: 11.5% vs. 1%) of G3–4 (CTCAE v3.0) ureteral stricture in patients with stage T3–4 with hydronephrosis at diagnosis vs. patients with stage T1. MV-CRA: HR = 15.0, *p* = 0.001.	Fokdal (2019) [88]
Low BMI	Higher risk of G3–4 (CTCAE v4.0) complications in patients with a BMI <18.5 vs. patients with a BMI >24.9. Complication rates (Chi-square tests): Bowel obstruction: 33.3% vs. 4.4%; *p* < 0.001. Lymphedema: 5.6% vs. 1.2%; *p* = 0.020. Enteritis: 16.7% vs. 13.6%; *p* = 0.030. MV-CRA: not significant.	Kizer (2011) [86]
	Higher risk of major rectal (10-year incidence: 5.4% vs. 2.8%) and small bowel (7.7% vs. 3.3%) complications in patients with a BMI <22 vs. patients with a BMI ≥22. MV-CRA: HR = 1.74, *p* = 0.010, and HR = 1.46, *p* = 0.04, respectively.	Eifel (2002) [87]
	A lower BMI was associated with a higher risk of G ≥3 bowel toxicity (CTCAE v3.0). MV-CRA: HR = 0.93, *p* = 0.014; attributable risk of 28% when patients with a BMI <25 were compared to patients with a BMI ≥25. Modeling of BMI using a spline model showed that per unit increase in BMI, the risk of G ≥3 bowel toxicity decreased by ~6%.	Laan (2017) [34]
	Higher risk (5-year actuarial rate 18.6% vs. 4%) of G≥3 bowel toxicity (CTCAE v3.0) in patients with BMI <18.5 vs. patients with BMI 18.5–24.9. MV-CRA: HR 13.99, *p* < 0.001.	Lee (2018) [99]
Obesity	Higher risk (10-year incidence: 3.7% vs. 2.8%) of major bladder complications in patients with a BMI ≥31 vs. patients with a BMI <31. MV-CRA: HR = 1.55, *p* = 0.050.	Eifel (2002) [87]
Race	Higher risk of major bladder (10-year incidence: 3.9% vs. 2.9%) and rectal (4.3% vs. 3.6%) complications in black women vs. white women. MV-CRA: HR = 1.89, *p* = 0.003, and HR = 1.73, *p* = 0.010, respectively. Lower risk (1.1% vs. 5.2%) of major small bowel complications in Hispanic women vs. white women. MV-CRA: HR = 0.27, *p* = 0.001.	Eifel (2002) [87]
Abdominal surgery	Higher risk of G ≥3 bowel toxicity (CTCAE v3.0) in patients with previous major abdominal surgery vs. patients without previous major abdominal surgery. MV-CRA: HR = 2.35, *p* = 0.013; attributable risk: 13%.	Laan (2017) [34]
	Higher risk (3-year actuarial rate: 15% vs. 8%) of major complications in patients who had prior surgery vs. patients who did not have prior surgery. MV-CRA: RR = 2.00, *p* = 0.0010.	Lanciano (1992) [85]
Staging laparotomy	Higher risk (3-year actuarial rate: 18% vs. 9%) of major complications in patients who had laparotomy for staging vs. patients who did not. Univariate analysis using the Mantel–Haenszel test: *p* = 0.0008. MV-CRA: not significant.	Lanciano (1992) [85]
	Higher risk (10-year actuarial rate: 14.5% vs. 3.7%) of small bowel obstruction in patients who underwent prior staging laparotomy vs. patients who did not. *p* < 0.0001 (Lee–Desu statistic).	Eifel (1995) [4]
Smoking	Higher risk (10-year incidence: 21.2 vs. 8.2%) of overall major complications in heavy smokers (≥1 packs/day) vs. non-smokers; MV-CRA: HR = 2.3, *p* < 0.0005. Most striking influence of heavy smoking was on the incidence of small bowel complications; MV-CRA: HR = 3.25, *p* < 0.0005. Light smokers (<1 pack/day) also had an increased risk of small bowel complications; MV-CRA: HR = 2.15, *p* = 0.006.	Eifel (2002) [87]
	Higher risk of G ≥3 bowel toxicity (CTCAE v3.0) in current smokers vs. patients that had never smoked or quit. MV-CRA: HR = 2.59, *p* = 0.001; attributable risk: 38%.	Laan (2017) [34]
	Higher risk of G3–4 (CTCAE v4.0) complications in current smokers vs. patients that never smoked. Complications included radiation enteritis/proctitis/cystitis, bowel obstruction, fistulae, and lymphedema. MV-CRA: HR = 1.78, *p* = 0.030.	Kizer (2011) [86]
	Higher risk of G ≥3 and G ≥2 (CTCAE v4.0) diarrhea in smokers vs. non-smokers. MV-CRA: HR = 3.02, *p* = 0.020, and HR = 1.47, *p* = 0.049, respectively. Higher risk of an EORTC “very much” rating for diarrhea in smokers vs. non-smokers. MV-CRA: HR = 1.92, *p* = 0.008.	Jensen (2021) [90]
Severe acute bowel toxicity	Higher risk of G ≥3 bowel toxicity (CTCAE v3.0) in patients with severe acute bowel toxicity vs. patients without severe acute bowel toxicity. MV-CRA: HR = 2.46, *p* = 0.010; attributable risk: 12%.	Laan (2017) [34]
Baseline diarrhea in the CTCAE	Higher risk of persistent G ≥1 diarrhea (CTCAE v4.0) in patients with baseline diarrhea vs. patients without baseline diarrhea. MV-BLRA: OR = 2.25, *p* = 0.011.	Jensen (2021) [90]
Baseline diarrhea on the EORTC scale	Higher risk of an EORTC “very much” rating for diarrhea in patients with baseline diarrhea vs. patients without baseline diarrhea. MV-CRA: HR = 7.29, *p* = <0.001. Higher risk of a persistent EORTC rating ≥ “quite a bit” of diarrhea in patients with baseline diarrhea vs. patients without baseline diarrhea. MV-BLRA: OR = 4.49, *p* < 0.001.	Jensen (2021) [90]
Hypertension	Higher risk of G ≥3 bowel toxicity (CTCAE v3.0) in patients with hypertension vs. patients without hypertension. MV-CRA: HR = 2.33, *p* = 0.009; attributable risk: 2%.	Laan (2017) [34]
Low SES	Higher risk of G ≥3 bowel toxicity (CTCAE v3.0) in patients with a low SES vs. patients with a high SES. MV-CRA: HR = 2.05, *p* = 0.012; attributable risk: 27%.	Laan (2017) [34]
Diabetes mellitus	Higher risk of persistent G ≥1 (CTCAE v4.0) diarrhea in patients with diabetes vs. those without. MV-BLRA: OR = 2.61, *p* = 0.004. Higher risk of a persistent EORTC rating ≥ “quite a bit” of diarrhea in patients with diabetes vs. those without. MV-BLRA: OR = 3.23, *p* = 0.017.	Jensen (2021) [90]
	**Prostate Cancer**	
Age	Higher risk of G ≥2 GU toxicity (CTCAE v3.0) in patients >69 years vs. patients ≤69 years. UV-CRA: RR = 2.5, *p* = 0.042. MV-CRA: not significant.	Ghadjar (2010) [82]
	Higher risk of proctitis in patients with “age + 1 year at baseline” vs. patients with “age at baseline”. MV-CRA (in 388 patients for whom dosimetry was available): HR = 1.06, *p* = 0.04. The HR represents the increase in risk per increased year of age at baseline.	Barnett (2011) [83]
Diabetes mellitus	Higher risk of G ≥2 GU and GI toxicity (RTOG) in diabetics vs. nondiabetics. GU—CRR-UVA: HR = 2.35, *p* = 0.043; CRR-MVA: not significant. GI—CRR-MVA: HR = 3.81, *p* = 0.008.	Hunter (2012) [91]
	Higher risk of G2 GU (5-year actuarial rate: 14% vs. 6%) and GI (28% vs. 17%) complications in diabetics vs. nondiabetics. Stepwise MV-CRA: *p* = 0.0097 and *p* = 0.0070, respectively.	Herold (1999) [92]
	Higher risk of “needing incontinence pads for rectal discharge” and “nocturia (≥4)” in diabetics vs. nondiabetics. Log-rank test: *p* = 0.020 and *p* = 0.010, respectively. MV-CRA: significant.	Peeters (2005) [6]
Pretreatment GI symptoms	Higher risk of G ≥2 and ≥3 GI toxicity in patients with pretreatment GI G ≥2 symptoms vs. patients with pretreatment GI G < 2 symptoms. CR-BM: HR = 4.1, *p* = 0.001, and HR = 8.4, *p* = 0.05, respectively. MV-CRA: significant. Patients with pretreatment GI G ≥2 symptoms had a higher incidence of two indicators: “pain/cramps/tenesmus requiring medication” (*p* = <0.001) and “use of incontinence pads because of blood/mucus/stool loss” (*p* = 0.020).	Peeters (2005) [6] *
Pretreatment GU symptoms	Higher risk (42% vs. 25%) of G ≥2 GU toxicity in patients with pretreatment GU G ≥2 symptoms vs. patients with pretreatment GU G < 2 symptoms. CR-BM: HR = 2.2, *p* < 0.001. MV-CRA: significant. Patients with pretreatment GU G ≥2 symptoms had a higher incidence of three indicators: “nocturia ≥4” (*p* = <0.001), “urinary obstruction requiring treatment’” (*p* = 0.020), and “urinary frequency” (*p* = 0.020).	Peeters (2005) [6]
	Higher risk of G ≥2 GU toxicity (CTCAE v3.0) in patients with pretreatment GU morbidity vs. those without. MV-CRA: RR = 9.4, *p* < 0.001.	Ghadjar (2010) [82]
Hormonal treatment	Higher risk of G ≥2 (34% vs. 25%) and ≥3 GU toxicity in patients with HT vs. patients without HT. CR-BM: HR = 2.2, *p* < 0.001, and HR = 2.3, *p* = 0.030, respectively. MV-CRA: significant. HT particularly resulted in a higher incidence of “nocturia ≥ 4” (*p* < 0.001).	Peeters (2005) [6]
Prior TURP	Higher risk of G ≥2 (41% vs. 25%) and ≥3 GU toxicity in patients with prior TURP vs. patients without prior TURP. CR-BM: HR = 1.7, *p* = 0.006, and HR = 3.1, *p* = 0.001. MV-CRA: significant. Most prominent indicators for GU toxicity in patients with prior TURP: “urinary obstruction” (*p* < 0.001) and “dysuria requiring drugs” (*p* = 0.050).	Peeters (2005) [6]
	Higher risk (78% vs. 53%) of G ≥2 toxicity (CTCAE v4.0) in patients with prior TURP vs. patients without prior TURP. MV-BLRA: OR = 3.6, *p* = 0.013.	Nuijens (2022) [28]
	Higher risk (5-year actuarial rate: 10% vs. 6%) of G ≥1 urinary incontinence in patients with prior TURP vs. patients without prior TURP. MV-CRA: RR = 1.8, *p* = 0.026.	Liu (2005) [94]
	Higher risk (5-year actuarial rate: 4% vs. 1%) of urethral stricture requiring dilatation in patients with prior TURP vs. patients without prior TURP. Mantel log-rank test: *p* = 0.010.	Sandhu (2000) [95]
	Higher risk of G ≥2 GU toxicity (CTCAE v3.0) in patients with TURP <12 months prior to radiotherapy vs. those without. UV-CRA: RR = 4.0, *p* = 0.007. MV-CRA: not significant.	Ghadjar (2010) [82]
Abdominal surgery	Higher risk of G ≥2 (28% vs. 15%) and G ≥3 GI toxicity in patients with previous abdominal surgery vs. patients without previous abdominal surgery. CR-BM: HR = 1.9, *p* < 0.001, and HR = 4.2, *p* = 0.010. MV-CRA: significant. Most prominent indicators for GI toxicity in patients with prior abdominal surgery: “bleeding requiring laser/transfusion” (*p* = 0.002), “use of incontinence pads” (*p* = 0.008), and “proctitis and use of steroids” (*p* = 0.050).	Peeters (2005) [6] *
	Higher risk of G2–3 rectal bleeding in patients with previous cholecystectomy vs. patients without previous cholecystectomy. MV-LRA: OR = 6.5, *p* = 0.002. Higher risk of G3 rectal bleeding in patients with previous appendectomy or cholecystectomy vs. those without. MV-LRA: OR = 5.9, *p* = 0.004, and OR = 5.5, *p* = 0.016, respectively.	Valdagni (2012) [96]
Acute GI toxicity	Higher risk of overall G ≥2 GI toxicity, “stool frequency ≥6×/day” and “pain/cramps/tenesmus requiring medication” in patients with acute G ≥2 GI toxicity vs. patients without acute GI toxicity. MV-CRA with clinical variables: HRs = 1.6, 2.9, and 1.9, respectively (*p* ≤ 0.010). MV-CRA including dosimetric parameters: not significant.	Peeters (2006) [93]
	Higher risk of overall G ≥2 GI toxicity in patients with acute mucous discharge (AMD) or acute proctitis vs. those without. MV-CRA: HRs = 1.8 and 1.7, respectively (*p* < 0.0001). Higher risk of “incontinence pads” and “stools ≥6/day” in patients with AMD or acute proctitis vs. those without. MV-CRA: HRs between 2.1 and 2.9, *p* = ≤0.004. For “stools ≥6/day”, the acute RTOG score was also a strong predictor: HR = 2.5, *p* = 0.0002. Higher risk of “intermittent bleeding” in patients with AMD or acute proctitis vs. those without. MV-CRA: HR = 1.6, *p* = 0.005, and HR = 1.5, *p* = 0.010, respectively.	Heemsbergen (2006) [23]
	Higher risk of proctitis and increased stool frequency in patients with acute bowel toxicity vs. those without. MV-CRA (in 388 patients for whom dosimetry was available): HR = 1.65, *p* = 0.050, and HR = 1.91, *p* = 0.003, respectively.	Barnett (2011) [83]
	Higher risk of G ≥2 fecal incontinence (actuarial and chronic) in patients with G ≥2 acute incontinence vs. those without. MV-LRA: OR = 6.9, *p* = 0.001, and OR = 4.34, *p* = 0.004, respectively.	Fellin (2009) [97]
	Higher risk of chronic G ≥2 rectal toxicity (CTCAE v2.0) in patients with G ≥2 acute rectal toxicity vs. those without. MV-CRA: HR = 2.6, *p* = 0.008.	Vargas (2005) [98]
	Higher risk (10-year incidence: 42% vs. 9%) of GI toxicity (CTCAE v3.0) in patients with G ≥2 acute GI toxicity vs. those without. Mantel log-rank test: *p* < 0.001. MV-CRA: HR = 6.95, *p* < 0.001.	Zelefsky (2008) [24]
Acute GU toxicity	Higher risk (5-year actuarial rate: 11% vs. 5%) of G ≥1 urinary incontinence in patients with G ≥2 acute GU toxicity vs. patients with G0–1 acute GU toxicity. MV-CRA: RR = 1.58, *p* = 0.002.	Liu (2005) [94]
	Higher risk of G ≥2 GU toxicity in patients with acute G2–3 GU toxicity vs. patients with acute G0–1 GU toxicity. UV-CRA: RR = 3.0, *p* = 0.020. MV-CRA: not significant.	Ghadjar (2010) [82]
	Higher risk of G ≥1 stress incontinence in prior TURP patients with acute G ≥2 urinary symptoms vs. prior TURP patients without acute urinary symptoms. MV-CRA: RR = 4.8, *p* = 0.010.	Sandhu (2000) [95]
	Higher risk (10-year incidence: 35% vs. 12%) of GU toxicity (CTCAE v3.0) in patients with G ≥2 acute GU toxicity vs. those without. Mantel log-rank test: *p* < 0.001. MV-CRA: HR = 3.22, *p* < 0.001.	Zelefsky (2008) [24]
Smoking	Higher risk of “dysuria requiring drugs” in smokers vs. non-smokers. Log-rank test: *p* = 0.020.	Peeters (2005) [6]
	Higher risk of rectal symptoms (Vaizey) in patients smoking five cigarettes/day vs. non-smokers. Kruskal–Wallis test: *p* < 0.010. Higher risk (26% vs. 10%) of urinary incontinence (NCI score ≥2) in smokers vs. non-smokers. Chi-square test: *p* < 0.010.	Thomas (2013) [89]
High BMI	Higher risk of rectal symptoms (Vaizey) in patients with a higher BMI: median Vaizey scores were 1.2, 2.6, and 3.4 for those with a BMI <24.9, of 25–30, and >30, respectively; *p* < 0.050. Higher risk of rectal bleeding (NCI score = 1) in patients with a higher BMI: incidences of 11%, 30%, and 33% for those with a BMI <24.9, of 25–30, and >30, respectively; *p* < 0.010. Higher risk of nocturia (NCI score ≥1) in patients with a higher BMI: incidences of 39%, 52%, and 55% for those with a BMI <24.9, of 25–30, and >30, respectively; *p* < 0.050. Either the Kruskal–Wallis or the Chi-square test was performed.	Thomas (2013) [89]
Physical inactivity	Higher risk of rectal symptoms (Vaizey) in inactive patients vs. those who took any sort of exercise (moderately inactive, moderately active, or active); *p* < 0.001. Higher risk (35% vs. 14%) of rectal bleeding in inactive vs. active patients; *p* < 0.050. Higher risk (16% vs. 5%) of urinary incontinence (NCI score >1) in inactive patients vs. those who took any sort of exercise; *p* < 0.010. Higher risk (56% vs. 32%) of nocturia (NCI score ≥1) in inactive patients vs. active patients; *p* < 0.010. Either the Kruskal–Wallis or the Chi-square test was performed.	Thomas (2013) [89]
Inflammatory bowel disease	Higher risk of fecal urgency in patients with IBD vs. patients without IBD. MV-CRA (in 388 patients for whom dosimetry was available): HR = 3.59, *p* = 0.008.	Barnett (2011) [83]

Abbreviations: G = grade; MV-LRA = multivariate logistic regression analysis; RR = relative risk; MV-CRA = multivariate Cox regression analysis; CTCAE v3.0 = Common Terminology Criteria for Adverse Events version 3; HR = hazard ratio; CTCAE v4.0 = Common Terminology Criteria for Adverse Events version 4; BMI = body mass index; EORTC = the European Organisation for Research and Treatment of Cancer; MV-BLRA = multivariate binary logistic regression analysis; OR = odds ratio; SES = socioeconomic status; GU = genitourinary; UV-CRA = univariate Cox regression analysis; GI = gastrointestinal; RTOG = Radiation Therapy Oncology Group; CRR-UVA = competing risk regression univariate analysis; CRR-MVA = competing risk regression multivariate analysis; CR-BM = Cox regression baseline model; HT = hormonal treatment; TURP = transurethral resection of the prostate; AMD = acute mucous discharge; NCI = National Cancer Institute; IBD = inflammatory bowel disease. * A later analysis, which included dosimetric parameters and had a longer follow-up, confirmed the associations of pretreatment GI symptoms and prior abdominal surgery with late GI toxicity [23].

**Table 2:** Candidate gene association studies on genetic variants and late side effects after radiotherapy in prostate and gynecological cancer patients.

Gene(s)	Treatment	N	Association	Ref.
*SOD2*, *XRCC1*, *XRCC3* (5 SNPs)	Prostate BT ± EBRT	135	Significant association with erectile dysfunction for *XRCC1* (rs25489). Significant association with late rectal bleeding grade 2 (RTOG) for *SOD2* (rs4880) alone and for a combination of *SOD2* and *XRCC3* (rs861539).	[139]
*ATM* (21 sequence variants representing 17 different alterations)	Prostate BT	37	Significant associations with late radiation toxicity for *ATM* sequence variants, particularly variants that encode for an amino acid substitution (i.e., missense mutations).	[136]
*ATM* (59 sequence variants, representing 25 different alterations)	Prostate BT ± EBRT	108	Significant association with late rectal bleeding/proctitis grades 1–2 (RTOG) for *ATM* sequence variants; for patients who receive the full prescription dose to either a low (<0.7 cm^3^) or moderate volume (0.7–1.4 cm^3^) of rectal tissue. Significant association with erectile dysfunction for *ATM* missense variants.	[137]
*ATM*, *TP53*, *MDM2* (7 SNPs)	Prostate EBRT	48	Significant association with chronic urinary toxicity grade ≥2 (RTOG) for one intronic *TP53* polymorphism (rs17883323).	[141]
*BRCA1*, *BRCA2*, *ESR1*, *XRCC1*, *XRCC2*, *XRCC3*, *NBN*, *RAD51*, *RAD52*, *LIG4*, *ATM*, *BCL2*, *TGFB1*, *MLH1*, *MSH6*, *ERCC2*, *XPF*, *NR3C1*, *CYP1A1*, *CYP2C9*, *CYP2C19*, *CYP3A5*, *CYP2D6*, *CYP11B2*, *CYP17A1* (49 SNPs)	Prostate EBRT	83	Significant univariate associations with late rectal or bladder toxicity grade ≥2 (RTOG) for *XRCC3* (rs1799794), *LIG4* (rs1805386), *MLH1* (rs1799977), and CYP2D6*4 (rs1800716). On a Cox multivariate analysis, *LIG4* (rs1805386), *ERCC2* (rs1052555), and CYP2D6*4 (rs1800716) showed significant associations with toxicity.	[138]
*XRCC1* (three SNPs)	Prostate EBRT	603	Significantly *decreased* risk of late bladder and/or rectal toxicity grade ≥2 (RTOG) in carriers of the Arg280His (rs25489) polymorphism (Kaplan–Meier analysis). This polymorphism remained a significant predictor in the multivariate analysis including clinical and dosimetric parameters.	[140]
*XRCC1*, *XRCC3*, *OGG1* (eight SNPs)	Cervical or endometrial EBRT + BT boost	62	Significant association with late toxicity grade ≥2 (CTCAEv3.0) for a *XRCC3* polymorphism in a noncoding sequence region (rs1799796). Significantly *decreased* risk of late toxicity grade ≥2 (CTCAEv3.0) in carriers of the *XRCC1* Arg194Trp (rs1799782) polymorphism. Patients with a combination of ≥2 (*XRCC1*) or ≥3 (*XRCC1* and *XRCC3*) risk alleles had significantly increased risks of grade ≥2 late toxicity, with ORs of 12.0 and 10.1, respectively.	[142]
*TGFB1* (six sequence variants of which five are SNPs)	Cervical or endometrial EBRT + BT boost	78	No significant associations with either late toxicity grade ≥2 or late toxicity grade ≥3 (CTCAEv3.0) for six *TGFB1* polymorphisms.	[143]

Abbreviations: N = number of patients; SNPs = single-nucleotide polymorphisms; BT = brachytherapy; EBRT = external beam radiotherapy; RTOG = Radiation Therapy Oncology Group; CTCAEv3.0 = Common Terminology Criteria for Adverse Events version 3; OR = odds ratio.

## Data Availability

No new data were created or analyzed in this study. Data sharing is not applicable to this article.

## Supplementary Materials

The following supporting information can be downloaded at https://www.mdpi.com/article/10.3390/curroncol32010047/s1. Table S1: Treatment and study information.
