# Supplementary material for: Towards Personalized Radiotherapy in Pelvic Cancer: Patient-Related Risk Factors for Late Radiation Toxicity
Source: Curr Oncol. 2025 Jan 17;32(1):47. doi: 10.3390/curroncol32010047 (PMC11763857; doi:10.3390/curroncol32010047)
Supplement: Supplementary file 1 [file curroncol-32-00047-s001.zip › curroncol-3337665-supplementary.pdf]

Supplementary Table S1. Treatment and study information

**Chen 2000**<br>Whole pelvis EBRT 40-44 Gy/20-22 Fx/4-5weeks. Boosted to 54-58 Gy with central shielding for patients with FIGO stage ≥IIb bilateral parametrial disease. Followed by HDRICB, 3-4 insertions at doses of 5-7.2 Gy (to Point A) at intervals of 1 week.<br>MV analysis: several treatment factors were considered, including cumulative rectal dose.<br>Study period: 1992 – 1995. N = 128<br>Retrospective. FU: median 43 M.<br>Grading: As reported by Esche *et al.*<br>G3 = fistulae, conditions requiring surgical correction, fatal complications; G2 = requiring hospitalization, transfusion or ≥6 M of medical therapy, continuous symptoms ≥6 M, those significantly changing the functional status, radiologically evident enteric or ureteric stenosis; G1 = no significant debility, lasted <6 M or were extremely intermittent.	**Lanciano 1992**<br>EBRT alone (whole pelvis or parametrial field only) or intracavitary radiotherapy alone. No further specifications regarding dose and Fx size.<br>MV analysis: several treatment factors were considered, including bladder and rectal doses.<br>Study period: 1973 and 1978. N = 1558<br>Retrospective. FU: median 43 M.<br>Grading: A major complication is one that necessitated hospitalization for treatment or surgery, or caused death.
**Fokdal 2019 –** EMBRACE studies<br>EBRT 45-50 Gy in 25-30 Fx, concomitant cisplatin, followed by IGABT.<br>MV analysis: several treatment factors were considered, including doses to OARs.<br>Study period: 1998 – 2012 (RetroEMBRACE) and 2008 – 2015 (EMBRACE I). N = 1860<br>Retrospective (n = 610) and prospective (n = 1250). FU: median 34 M.<br>Grading: CTCAE v3.0.	**Kizer 2011**<br>Standard EBRT for cervical cancer at Barnes-Jewish Hospital/Washington University School of Medicine in St. Louis, Missouri. 128 (32%) received IMRT and 274 (68%) received conventional RT. 399 (99%) and 288 (92%) also received cervical BT and concurrent chemotherapy, respectively.<br>MV analysis: unclear whether treatment factors were considered, dose to OARs most likely not included.<br>Study period: 1998 – 2008. N = 404<br>Retrospective. FU: mean 47.2 M.<br>Grading: CTCAE v4.0.
**Eifel 2002**<br>3296 (94%) were treated with EBRT followed by intracavitary BT. 113 (3%) were treated with BT alone. 80 (2%) were treated with EBRT alone. Most patients (96%) received an EBRT dose of 40-45 Gy to the central pelvis in Fx of 1.8-2 Gy.<br>MV analysis: included treatment factors were dose of radiation delivered to the central pelvis (EBRT) and history of transperitoneal lymph node dissection<br>Study period: 1960 – 1994. N = 3489<br>Retrospective. FU: median 85 M.<br>Grading: A major complication is one that required hospitalization, surgery, or resulted in death. Severe	**Laan 2017**<br>Pelvic EBRT and intracavitary BT. The most frequently prescribed EBRT dose was 46 Gy in 2 Gy/Fx. Concurrent chemoradiation (weekly cisplatin) was introduced in 1999.<br>MV analysis: several treatment factors were considered, such as EBRT field and technique, but not doses to OARs.<br>Study period: 1992 – 2013. N = 515 (389 for MV)<br>Retrospective. FU: median 78 M.<br>Grading: CTCAE v3.0.

complications, including those needing transfusion, chronic narcotic use, or causing uncontrolled severe diarrhea or bladder symptoms, were also classified as major.	
**Lee 2018**<br>Pelvic EBRT 45-50.4 Gy delivered as IMRT in 1.8 Gy/Fx. Involved lymph nodes were boosted to 59.4 Gy. Concomitant cisplatin in 208 (85%). HDRICB either 1x/week concurrently with EBRT or 2x/week after EBRT; 6 insertions at doses of 5 Gy (to Point A).<br>MV analysis: some treatment factors were considered, including bowel bag V45.<br>Study period: 2004 – 2015. N = 245<br>Retrospective. FU: median 63 M.<br>Grading: CTCAE v3.0.	**Eifel 1995**<br>1679 (94%) were treated with EBRT followed by intracavitary BT. 80 (5%) were treated with BT alone. 25 (1%) were treated with EBRT alone. 234 (13%) underwent adjuvant hysterectomy, in these patients the intracavitary irradiation was usually reduced by 25%: one 72-h implant after 40 Gy to the whole pelvis. EBRT in 2 Gy/Fx.<br>MV analysis: not performed.<br>Study period: 1960 – 1989. N = 1784<br>Retrospective. FU: median 54 M.<br>Grading: A major complication is one that required hospitalization, transfusion, or an operation or caused severe symptoms or the patient’s death.
**Jensen 2021 -** EMBRACE I<br>EBRT 45-50 Gy in 25-30 Fx delivered as IMRT/VMAT or 3DCRT with concomitant weekly chemotherapy (in 95% of the patients), and IGABT according to the GEC-ESTRO guidelines.<br>MV analysis: several treatment factors were considered, including doses to OARs, such as rectum D2cc ($EQD2_3$).<br>Study period: 2008 - 2015.<br>N = 1199 (PRO available in 900)<br>Prospective. FU: median 48 M.<br>Grading: CTCAE v3.0 & EORTC (QLQ-C30).	**Ghadjar 2010**<br>All patients were treated with 80 Gy in 2 Gy/Fx using IMRT.<br>MV analysis: some treatment factors were considered, including rectal V75 and V47.<br>Study period: 2004 – 2008. N = 102<br>Retrospective. FU: median 39 M.<br>Grading: CTCAE v3.0.
**Barnett 2011** - Medical Research Council RT01 trial<br>394 and 394 were treated with 74 Gy and 64 Gy, respectively. 2 Gy/Fr, using CRT. Initial 64 Gy: three- or four-field plans. Four- or six-field plans were used for the additional 10 Gy for those patients who were randomized to receive 74 Gy.<br>MV analysis: several treatment factors were considered, including several rectal dosimetric parameters such as rectal V40 and V70.<br>Study period: 1998 – 2002. N = 788<br>Prospective. FU: ≥24 M.<br>Grading: LENT/SOMA; Prostate Cancer Index of the Royal Marsden Hospital and the University of California, LA.	**Hunter 2012**<br>116 (24%) were treated with interstitial BT, 195 (40%) with RP, and 172 (36%) with RT. 11/172: CRT, 78 Gy in 2 Gy/Fr. 161/172: five-field IMRT, 70 Gy in 2.5 Gy/Fr.<br>MV analysis: only type of treatment (EBRT, BT, RP) was considered, not dose to OARs.<br>Study period: 1999. N = 483<br>Retrospective. FU: median M.<br>Grading: RTOG.
**Herold 1999**	**Peeters 2005** - Dutch dose-escalation trial

100% were treated using 3DCRT. Doses to the center of the prostate ranged from 6211 to 8074 cGy, median dose 7211 cGy. Unspecified Fx size.<br>MV analysis: some treatment factors were considered, such as field size and target dose, but not doses to OARs.<br>Study period: 1989 – 1996. N = 944<br>Retrospective. FU: median 36 M.<br>Grading: RTOG and modified LENT scales.	320 (50%) and 323 (50%) were treated with 68 Gy and 78 Gy, respectively. 2 Gy/Fr, using 3DCRT. The boost of 10 Gy was given sequentially with 3DCRT, except for 41 patients, where a simultaneous integrated boost was given using IMRT.<br>MV analysis: only randomization arm and dose-volume group (defined according to estimated risk of seminal vesicles involvement) were considered, not dose to OARs.<br>Study period: 1997 – 2003. N = 643<br>Prospective. FU: median 31 M.<br>Grading: Slightly adapted RTOG/EORTC scales and 12 specific complications ('indicators').
**Nuijens 2022**<br>170 (96%) were treated with 77 Gy. 2.2 Gy/Fr, using IMRT.<br>MV analysis: several treatment factors were considered, including doses to OARs, such as bladder V70 and rectum V70.<br>Study period: 2009 – 2013. N = 179<br>Prospective. FU: median 31 M.<br>Grading: CTCAE v4.0.	**Liu 2005**<br>All received between 50 and 72 Gy in 20-37 Fx (median isocentric dose 66 Gy; median of 33 Fx). A box technique was used. Three-dimensional planning since 1998.<br>MV analysis: only radiation dose and Fx size were considered, not dose to OARs.<br>Study period: 1994 – 2000. N = 1158<br>Prospective. FU: median 52 M.<br>Grading: Modified RTOG/SOMA scale: G1 = occasional use of pads; G2 = intermittent use of pads; G3 = persistent use of pads; G4 = permanent catheter.
**Sandhu 2000**<br>Median radiation dose prescribed to the PTV was 75.6 Gy (range: 64.8 – 81 Gy). 3DCRT.<br>MV analysis: only radiation dose (<75.6 Gy vs. ≥75.6 Gy) was considered, not dose to OARs.<br>Study period: 1988 – 1997.<br>N = 1100 (120 had prior TURP)<br>Prospective. FU: median 51 M.<br>Grading: RTOG; LENT/SOMA for degree of stress incontinence.	**Valdagni 2012** - AIROPROS 0102<br>Treatment dose ≥70 Gy, in 1.8-2 Gy/Fx, using 3DCRT.<br>MV analysis: several rectal dosimetric parameters were considered, such as mean rectal dose and V70.<br>Study period: 2002 – 2004. N = 718<br>Prospective. FU: 36 M for all patients.<br>Grading: Self-reported questionnaire, answers were used to classify symptoms according to SOMA/LENT.<br>Focus: grade 2-3 fecal incontinence and late rectal bleeding.
**Peeters 2006** - Dutch dose-escalation trial<br>326 (50%) and 330 (50%) were treated with 68 Gy and 78 Gy, respectively. 2 Gy/Fx, using 3DCRT. The boost of 10 Gy was given sequentially with 3DCRT, except for 41 patients, where a simultaneous integrated boost was given using IMRT.<br>MV analysis: several dosimetric parameters for different OARs were considered, including rectal V5-V70, maximum, and mean dose.	**Heemsbergen 2006** - Dutch dose-escalation trial<br>275 (50%) and 278 (50%) were treated with 68 Gy and 78 Gy, respectively. 2 Gy/Fx, using 3DCRT.<br>MV analysis: relevant dose parameters from a previous study were considered, including anorectal V65 and mean dose to the anorectum.<br>Study period: 1997 – 2003. N = 553<br>Prospective. FU: median 44 M.<br>Grading: Acute: RTOG/EORTC and 2 specific toxicity

Study period: 1997 – 2003. N = 656<br>Prospective. FU: median 44 M.<br>Grading: Slightly adapted RTOG/EORTC scales and 5 specific complications (‘indicators’).	indicators. Late: RTOG/EORTC and 5 specific toxicity indicators.
**Fellin 2009** - AIROPROS 0102<br>Treatment dose ≥70 Gy, in 1.8-2 Gy/Fx, using 3DCRT.<br>MV analysis: several rectal dosimetric parameters were considered, such as mean rectal wall dose and rectal V70.<br>Study period: 2002 – 2004. N = 718<br>Prospective. FU: 36 M for all patients.<br>Grading: Self-reported questionnaire, answers were used to classify symptoms according to SOMA/LENT.<br>Focus: grade 2-3 fecal incontinence and late rectal bleeding.	**Vargas 2005**<br>Median treatment dose of 75.6 Gy. 1.8 Gy/Fx, using 3DCRT.<br>MV analysis: several rectal dosimetric parameters were considered, such as mean rectal dose and V70.<br>Study period: 1999 – 2002. N = 331<br>Prospective. FU: median 18 M.<br>Grading: CTCAE v2.0.
**Zelefsky 2008**<br>Treatment dose range: 66 to 81 Gy. 1.8 Gy/Fx. 3DCRT or IMRT. All IMRT patients (n = ?) received 81 Gy. 358 (23%), 472 (30%), and 741 (47%) patients received ≤70.2 Gy, 75.6, and 81 Gy, respectively.<br>MV analysis: only radiation dose (<81 Gy vs. 81 Gy = 3DCRT vs. IMRT) was considered, not dose to OARs.<br>Study period: 1988 – 2000. N = 1571<br>Retrospective. FU: median 10 years.<br>Grading: CTCAE v3.0.	**Thomas 2013**<br>Treatment with one of five radical regimens in use at Addenbrooke’s Hospital: 64 Gy in 32 Fx, 55 Gy in 20 Fx, 74 Gy in 34 Fx, 60 Gy in 20 Fx, or 57 Gy in 18 Fx. Either CRT, IMRT, or IGRT was used.<br>MV analysis: treatment factors (including doses to OARs) were not considered.<br>Study period: 2000 – 2010. N = 440<br>Retrospective. FU: median 43 M.<br>Grading: Questionnaires: NCI common symptoms scores for rectal bleeding, nocturia, urinary incontinence and erectile function, and the Vaizey Rectal Symptoms Score.

Abbreviations: EBRT = external beam radiotherapy; Fx = fraction; FIGO = Fédération Internationale de Gynécologie et d'Obstétrique; HDRICB = high dose rate intracavitary brachytherapy; MV = multivariate; N = number of patients; FU = follow-up; G = grade; M = months; IGABT = image guided adaptive brachytherapy; OARs = organs at risk; CTCAE vX = Common Terminology Criteria for Adverse Events version X; IMRT = intensity modulated radiotherapy; RT = radiotherapy; BT = brachytherapy; VMAT = volumatic modulated arc therapy; 3DCRT = 3-dimensional conformal radiotherapy; GEC-ESTRO = Groupe Européen de Curiethérapie - European SocieTy for Radiotherapy & Oncology; D2cc = minimal dose to the most exposed 2 cc of the respective organ; $EQD2_3$ = equieffective dose in 2 Gy per fraction, with alpha/beta ratio of 3 Gy; PRO = patient-reported outcome; EORTC = European Organization for Research and Treatment of Cancer; QLQ-C30 = Core Quality of Life Questionnaire; Rectal VX = volume (%) of rectum receiving ≥X Gy; CRT = conformal radiotherapy; LENT = Late Effects Normal Tissues; SOMA = Subjective, Objective, Management, and Analytic; RP = radical prostatectomy; RTOG = Radiation Therapy Oncology Group; Bladder VX = volume (%) of bladder receiving ≥X Gy; PTV = planning target volume; TURP = transurethral resection of the prostate; anorectal VX = volume (%) of anorectum receiving ≥X Gy; IGRT = image-guided radiotherapy; NCI = national cancer institute.
